# Fabry-Perot Interferometric High-Temperature Sensing Up to 1200 °C Based on a Silica Glass Photonic Crystal Fiber

**DOI:** 10.3390/s18010273

**Published:** 2018-01-18

**Authors:** Haihu Yu, Ying Wang, Jian Ma, Zhou Zheng, Zhuozhao Luo, Yu Zheng

**Affiliations:** National Engineering Laboratory for Fiber Optic Sensing Technology, Wuhan University of Technology, Wuhan 430070, China; hhyu@whut.edu.cn (H.Y.); wang_ying@whut.edu.cn (Y.W.); jeromema@whut.edu.cn (J.M.); luojianlihun@whut.edu.cn (Z.Z.); luozz@whut.edu.cn (Z.L.)

**Keywords:** optical fiber sensor, high temperature, photonic crystal fiber, Fabry-Perot interferometer

## Abstract

A Fabry-Perot interferometric sensor for temperature measurement was fabricated based on a silica glass solid-core photonic crystal fiber with a central air-bore. By splicing a stub of photonic crystal fiber to a standard single-mode fiber, an intrinsic Fabry-Perot cavity was formed inside the photonic crystal fiber. Sensing experiment results show that the sensor can work stably for a consecutive 24 h under temperatures up to 1100 °C, and the short-term operation temperature can reach as high as 1200 °C (<30 min). In the measurement range of 300–1200 °C, the temperature sensitivity of the peak wavelength shift can reach as high as 15.61 pm/°C, with a linearity of 99.76%. The presented interferometric sensor is compact in size and possesses advantages such as an extended working range and high sensitivity, showing promising application prospects.

## 1. Introduction

High-temperature sensing has a wide range of applications in many fields such as metallurgy, energy, and the chemical and aerospace industries [1]. Thermocouples and infrared thermometers are two widely used high-temperature sensors. However, both schemes have drawbacks. For example, the wire of a thermocouple is easily corroded in a corrosive atmosphere, leading to a short service life and a high cost [2,3]. The measured result of the infrared thermometer is influenced by many factors such as background radiation, measuring distance, and gas composition, which amplify sensing errors [4,5]. With many advantages such as high sensitivity, compact size, immunity to electromagnetic interferences, and durability amid harsh environments [6], optical fiber sensing technology has developed rapidly, and various optical fiber approaches for high-temperature measurement have also been reported [1,7,8,9,10,11,12,13]. Among these optical fiber high-temperature sensors, regenerated fiber Bragg gratings (RFBGs) have received much attention. In a flammability test complying with ISO standards, the maximum temperature measured with RFBGs can reach ~970 °C [10]. High-temperature measurement from 600 to 1000 °C was also achieved using RFBGs inscribed on a boron/germanium co-doped fiber [11]. However, the working range of RFBGs is strongly related to the doped components and concentration, and RFBGs can be erased when beyond the glass-softening temperature of the doped core. Sapphire fiber sensor is another prospective candidate because sapphire has a high melting point as well as stable physical and chemical properties. Based on a sapphire fiber blackbody cavity, high-temperature measurement up to 1880 °C has been realized [13]. However, the processing of sapphire fiber and its coupling with conventional fibers are much more difficult than those of the silica glass fiber-based sensors. High-temperature sensors in various configurations based on conventional silica glass step-index fibers have also been demonstrated. Micco et al. demonstrated an interferometric sensor consisting of a thin layer of Si/H, which acts as a Fabry-Perot (F-P) cavity, embedded inside a single-mode fiber (SMF) by fusion splicing approach [14]. Benefiting from the high thermo-optic coefficient of the Si/H layer, the sensitivity reaches as high as 106 pm/°C in the range of 120–400 °C. Nguyen et al. reported a Mach-Zehnder interferometer based on a multimode–singlemode–multimode fiber cascaded configuration [15]. The unique structure induces interference between cladding and core modes propagated along the SMF. The temperature measurement limit can exceed 900 °C with a high sensitivity of 88 pm/°C. However, because the core of conventional step-index fibers is doped with other materials such as germanium, the glass-softening temperature is lower than that of pure silica glass and hence the upper measurement temperature is limited.

Photonic crystal fiber (PCF) is a special kind of optical fiber with complex refractive index distribution over the cross section, which is usually realized by the introduction of cladding air holes in specially designed arrangements [16,17]. In contrast to the conventional step-index fibers with doped cores, PCFs can be made from pure silica glass, which benefits from the unique light guiding mechanism and can work in a high-temperature environment until it is close to the softening temperature of silica glass. In view of this unique advantage, high-temperature sensing using PCFs has attracted increased research passions. In recent years, various interferometric high-temperature sensors based on diverse PCF structures have been proposed [18,19,20,21,22]. For example, using an F-P sensor fabricated from a polarization-maintaining PCF, temperature sensing in the range from 33 to 600 °C was realized with a sensitivity of 13.8 pm/°C [19]. Based on a stable PCF-based two-mode interferometric sensor, temperatures as high as 1000 °C can be measured with a sensitivity of 8.3 pm/°C [20]. An F-P interferometric high-temperature sensor based on a cascaded PCF-hollow fiber-SMF structure was designed by Choi et al., whose measurement could also reach 1000 °C [21]. By means of a multimode interference within a suspended-core PCF, high-temperature measurement up to 1100 °C with a sensitivity of 11 pm/°C was realized [22]. For high-temperature measurements, a higher upper-temperature limit and sensitivity are always expected, which will contribute to a wider working scope and higher temperature resolution [8]. The viable approaches to improve the performance of sensors include the design of novel sensing structures, the adoption of materials with a higher glass-softening temperature, thermo-optic coefficient, and thermo-expansion coefficient.

In this paper, we present an interferometric high-temperature sensor made of a pure silica glass solid-core PCF with a central air-bore. The sensor head was fabricated by fusion splicing a stub of PCF to an SMF to form a Fabry-Perot cavity inside the PCF. A theoretical analysis was conducted to study the temperature sensing mechanism of the F-P interferometric sensor. Experimental results show that, in a wide range of 300–1200 °C, the temperature measurement has a high sensitivity of 15.61 pm/°C with a linearity of 99.76%. The sensor is capable of stable operation during continuous heating at 1100 °C for 24 h, and the short-term operation temperature can reach as high as 1200 °C. To our knowledge, this sensor has an upper limit and sensitivity superior to many reported interferometric optical fiber temperature sensors, thereby showing promising application prospects.

## 2. Sensor Fabrication

**Figure 1:**
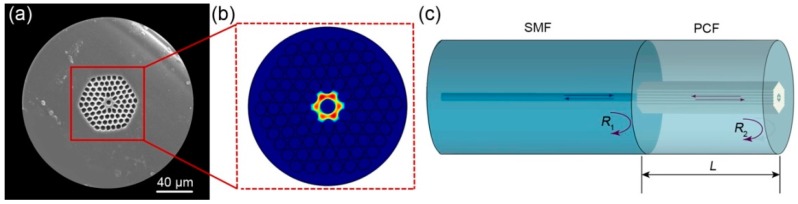
(**a**) SEM image of the solid-core photonic crystal fiber with a central air-bore; (**b**) electric field distribution of the fundamental mode; (**c**) structure schematic of the Fabry-Perot interferometric sensor head.

Figure 1a shows the scanning electron microscope (SEM) image of the solid-core silica glass photonic crystal fiber with a central air-bore. The PCF was drawn in our customized drawing tower, using the “stack-and-draw” method [23]. The high-purity fused silica capillaries and rods used to fabricate the PCF were purchased from Heraeus, Germany, which have a maximum allowable working temperature of 1200 °C and a higher softening temperature. The outer diameter of the fabricated PCF is 200 μm. The diameter of the central core and central air-bore is 9.00 μm and 4.82 μm, respectively. The cladding air-hole pitch is 4.75 μm and the cladding air-filling-fraction is 58%. Owing to the high pressure applied during the drawing process, the cladding air holes have a certain degree of distortion. Figure 1b shows the simulated electric field distribution of the fundamental mode (LP_01_) at 1530 nm, based on the finite element method (FEM) using the commercial software COMSOL Multiphysics. The simulation model was directly digitalized from the SEM image. At the periphery of the cladding, scattering boundary conditions were employed to eliminate boundary reflections. It can be seen that air holes in the cladding significantly reduce the cladding index, resulting in a higher index of the fiber core so that the confinement of light is based on the total internal reflection (TIR). Unlike conventional solid-core PCFs, the introduction of the central air-bore squeezes the mode field into six symmetric lobes. Since each lobe has a much smaller mode field area, the energy distribution is more concentrated, so the generation and transmission of high-order modes can be effectively suppressed, leading to homogeneous and strong interference spectra in the following F-P interferometric sensor.

The structure schematic of the F-P sensor is shown in Figure 1c. The sensor was fabricated by splicing an SMF with a short piece of the PCF. In order to facilitate the splicing process and enhance the bonding strength between the SMF and PCF, the outer diameter of the PCF was etched to 125 μm (the same with that of the SMF) by hydrofluoric acid before splicing. In case of the infiltration of hydrofluoric acid into the inner air holes, the two end faces of the PCF were sealed by UV-hardening glue in advance. After splicing, the PCF was cleaved to a certain length *L*. At the junction between the PCF and SMF, a collapsed region was formed due to the strong electric arc discharge. The collapse leads to a great increment of the reflectivity so that the collapsed region has a reflectivity *R*_1_. The smooth free end face is perpendicular to the fiber axis and acts as another reflector *R*_2_. Owing to Fresnel reflection, the collapsed region and the free end face form the Fabry-Perot interferometer together.

In view of the unique mode shape of the PCF with a central air-bore, the coupling efficiency between the SMF and PCF was calculated. Without considering the loss from Fresnel reflection, the minimum splice loss *η* between the SMF and PCF induced by mode field mismatch and center offset of mode field can be calculated by the following equation [24]:

(1) $$\eta \;=-10\log\left[\left(\frac{2\omega_{1}\omega_{2}}{\omega_{1}{}^{2}+\omega_{2}{}^{2}}\right){}^{2}\times \exp(\frac{-2d^{2}}{\omega_{1}{}^{2}+\omega_{2}{}^{2}})\right]$$

where 2*ω*_1_ and 2*ω*_2_ are the mode field diameters of the two spliced fibers and *d* denotes the center misalignment between the two mode fields. Since the mode field of PCF is squeezed into six symmetric lobes, with each lobe having a Gaussian-like profile, the coupled power is calculated by summing the contributions from each individual lobe, reminiscent of a “six-core” fiber. The mode field diameter of the SMF 2*ω*_1_ is 9 μm, and 2*ω*_2_ is ~3.234 μm for each lobe, calculated using the FEM. The center offset *d* is ~4.027 μm. Based on Equation (1), the coupled power into each lobe is calculated to be ~9.8% and into total six lobes is ~58.8%, corresponding to a minimum splice loss *η* ~2.3 dB. Therefore, most of the light from the SMF can be theoretically coupled into the PCF.

## 3. Theoretical Analysis

**Figure 2:**
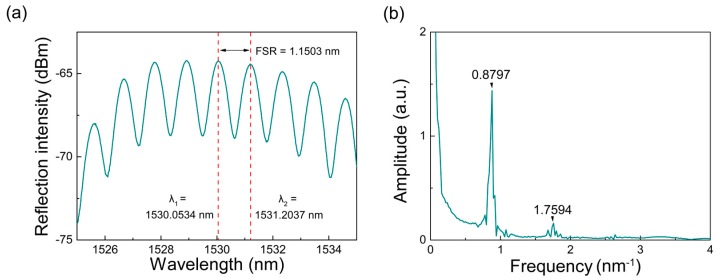
(**a**) Reflected interferometric pattern measured at 27 °C (room temperature); (**b**) corresponding FFT spectrum of the interferometric pattern.

When connecting the fabricated F-P interferometer to a C-band ASE light source (A20120003, Wuhan Oudi, Wuhan, China) and an optical spectrum analyzer (OSA; AQ6370B, Yokogawa, Tokyo, Japan) through a fiber circulator, regular and stable interference patterns can be observed, as shown in Figure 2a. The interference fringes have a maximum reflection intensity of ~64 dBm and a contrast of ~4.6 dBm. The free spectral range (FSR) of the interference fringes is 1.1503 nm at a 1530 nm wavelength. According to the following equation, FSR has a direct correlation with the wavelength *λ*, the effective refractive index of the interference mode *n*_eff_ and the F-P cavity length *L* [19]:

(2) $$\mathrm{FSR}=\frac{\lambda^{2}}{2n_{\mathrm{eff}}L}.$$

Figure 2b shows the fast Fourier transform (FFT) spectrum of the interference pattern. There is only one main peak and a faint frequency-doubled peak in the FFT spectrum, indicating the high homogeneity that no high-order mode participates in the interference. Based on the FEM, the effective refractive index of the fundamental mode *n*_eff_ is calculated to be 1.42287 at 1530 nm. Using Equation (2), the cavity length *L* of the fabricated interferometer is calculated to be 715 μm.

Let *I*_1_ and *I*_2_ be the intensities of the two reflected beams, the spectral intensity of the F-P interferometer is given by [19]

(3) $$I=I_{1}+I_{2}+2\sqrt{I_{1}I_{2}}\cos\varphi$$

with a phase difference of

(4) $$\varphi =4\pi n_{\mathrm{eff}}L/\lambda .$$

Note that the phase difference between two adjacent peaks satisfies the interference condition *ϕ* = 2*m*π (*m* is the integer), and Equation (4) can be rewritten as

(5) $$\frac{d}{\lambda }=m$$

where *d* = 2*n*_eff_*L* is defined as the optical path difference (OPD). If the wavelengths of the two adjacent resonance peaks are *λ*_1_ and *λ*_2_, respectively, the OPD can be obtained with the following [25]:

(6) $$d=\frac{\lambda_{1}\lambda_{2}}{2(\lambda_{2}-\lambda_{1})}.$$

Owing to the thermo-optic and thermal-expansion effects, the refractive index of pure silica glass and cavity length change with the variation of ambient temperature. We assume the change rate of the effective refractive index of the fundamental mode keeps step with the change rate of the refractive index of pure silica glass, and the change of OPD with temperature is approximated by

(7) $$\Delta d=2L(\delta_{T}+n_{\mathrm{eff}}\alpha_{T})\Delta T$$

where *δ*_T_ = 8.5 × 10^−6^/°C (Corning 7980 fused silica [26]) and *α*_T_ = 5.4 × 10^−7^/°C (Heraeus fused silica) are the thermo-optic and thermal-expansion coefficients of pure silica glass, respectively. Finally, the wavelength shift of the resonance peak with temperature (sensitivity) is given by

(8) $$\Delta \lambda =\frac{\Delta d}{m}=\frac{2}{m}L(\delta_{T}+n_{\mathrm{eff}}\alpha_{T})\Delta T.$$

Since the thermo-optic coefficient is more than ten times larger than the thermal-expansion coefficient, the thermo-optic effect mainly drives the wavelength shift. As shown in Figure 2a, two adjacent resonance peaks are located at 1530.0534 nm and 1531.2037 nm, which are marked as *λ*_1_ and *λ*_2_, respectively, and are used to determine the OPD based on Equation (6) and the integer *m* based on Equation (5). The integer *m* is calculated to be 665 at 1530 nm. Based on Equation (8), the theoretical temperature sensitivity of the Fabry-Perot interferometric sensor is calculated to be 19.93 pm/°C.

## 4. Experimental Results and Discussion

**Figure 3:**
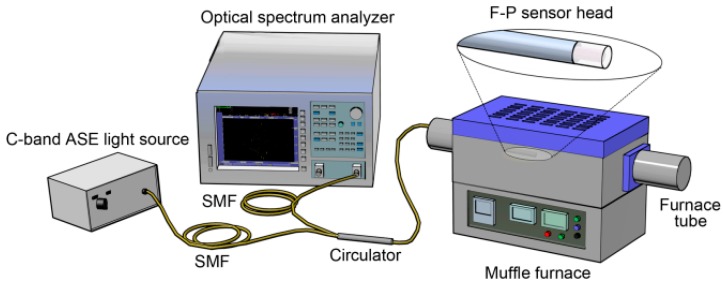
Experimental setup for temperature measurement.

The experimental setup for high-temperature sensing is shown in Figure 3. The F-P sensor head was inserted into a quartz tube for protection and together put into a muffle furnace (GSL-1600X, Hefei Kejing, Hefei, China) with a maximum working temperature of 1600 °C. The incident light was emitted from the C-band light source, and the reflected interferometric signals from the sensing head were collected by the OSA through an optical fiber circulator. Due to the inaccurate temperature control of the muffle furnace at low temperature, we began the measurement from 300 °C. The temperature was increased from 300 to 1200 °C in a step of 100 °C. Each step had a 20 min temperature increase and a 10 min temperature holding. The data were recorded when the temperature became completely stable.

**Figure 4:**
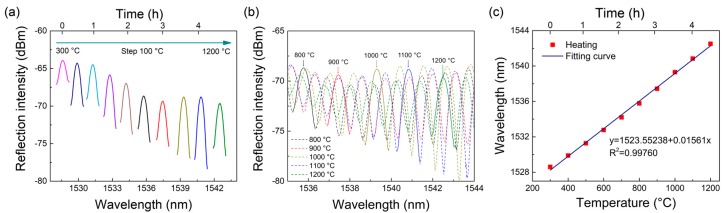
(**a**) Shift of a single reflection peak (located at 1528.60 nm at 300 °C) with the rising of temperature from 300 °C to 1200 °C; (**b**) shift of full spectra with the rising of temperature from 800 to 1200 °C; (**c**) linear fitting curve between peak wavelength and temperature from 300 to 1200 °C.

The interference spectra at different temperatures are shown in Figure 4a,b. Because of the wide temperature variation range, the interference spectra overlapped to some extent. For a clear illustration, Figure 4a only plots the shift of the interferometric peak at 1528.60 nm (300 °C). The shift of full spectra in the temperature range of 800–1200 °C is shown in Figure 4b. With increasing temperature, the interferometric spectra shift regularly toward the long-wavelength direction. The wavelength shift Δ*λ* of the interferometric peak at 1528.60 nm with temperature variation Δ*T* from 300 to 1200 °C can be well expressed by the following equation:

(9) Δλ=1523.55238+0.01561ΔT

of which the linearity is up to 99.76%. In the wide range of 300–1200 °C, the high linear coefficient guarantees the sensing accuracy. The slope of Equation (9) represents the temperature sensitivity, which reaches as high as 15.61 pm/°C. This experimental sensitivity is lower than the theoretical result of 19.93 pm/°C, which is probably because the thermo-optic coefficient *δ*_T_ of the fabricated PCF is lower than the value used in the theoretical calculation. Nevertheless, the experimental sensitivity is much higher than that of many reported optical fiber high-temperature sensors [18,19,20,22].

**Figure 5:**
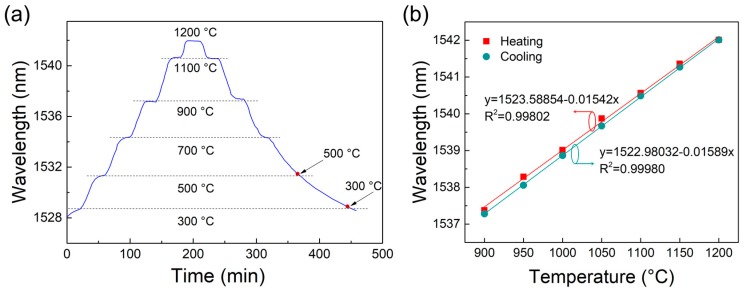
(**a**) Repeatability test from 300, to 1200, and back to 300 °C. The peak wavelengths when cooling through 500 °C and 300 °C are marked in red points; (**b**) repeatability test and linear fitting curve in the high-temperature range from 900, to 1200, and back to 900 °C.

The repeatability of the sensor was examined. The sensor was heated from 300 to 1200 °C and then cooled to 300 °C. The set heating and cooling rate were both 10 °C/min. At 300, 500, 700, 900, 1100, and 1200 °C, the temperature was maintained for 20 min. The shift of the peak at 1528.60 nm (300 °C) with time is shown in Figure 5a. It should be noted that, when the temperature drops to below ~700 °C, the temperature decreasing rate becomes much slower and requires a longer period of time to reach the set temperature; hence, when reaching 500 and 300 °C (the red points marked in Figure 5a), the temperature inside the muffle furnace was not maintained but continues to decline. It can be seen that, although some time is needed to stabilize the interference spectra, the peak wavelength can basically overlap when heating and cooling through the same temperature after being stable. We further carried out the repeatability test in the high-temperature range between 900 and 1200 °C in a shorter step of 50 °C. The heating and cooling rate remained 10 °C/min and the temperature was kept unchanged for 10 min. As shown in Figure 5b, the sensitivity when heating and cooling between 900 and 1200 °C is 15.42 pm/°C and 15.89 pm/°C, which are very close to the 15.61 pm/°C sensitivity in the full working range from 300 to 1200 °C. The maximum wavelength difference between the heating and cooling curves is 0.229 nm at 950 °C, which equals to a measured temperature difference of a maximum of ~15 °C. The error is acceptable, and the repeatability of the sensor can basically be guaranteed.

**Figure 6:**
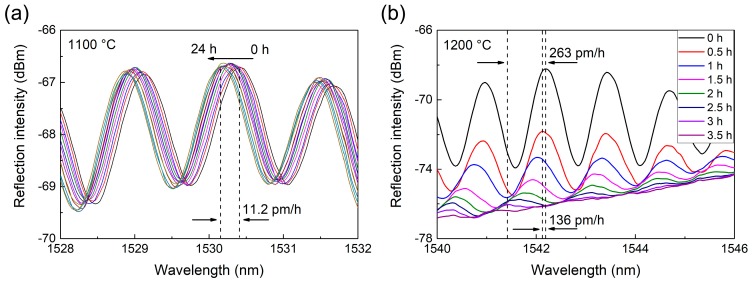
(**a**) Stability test of the sensor over 24 h at constant 1100 °C; (**b**) stability test at constant 1200 °C. The spectral contrast gradually decreases and finally disappears after 3 h.

Lastly, the stability of the sensor was measured. The sensor was kept at a constant temperature for a period of time, and the peak wavelength shift was monitored. Figure 6a shows the interference spectra during 24 h of continuous heating at 1100 °C. The spectral contrast remains stable, and peak wavelength slightly shifts at a rate of ~11.2 pm/h, corresponding to a slightly increased measuring error of ~0.7 °C/h. Therefore, the sensor has good stability and is able to continuously operate at 1100 °C with fine accuracy. The heating temperature was then further increased to 1200 °C, and the interference spectra are plotted in Figure 6b. At this higher temperature, the spectra became unstable and constantly shifted at a high rate of ~263 pm/h, indicating that the measuring error increased at a rate of ~16.8 °C/h. More importantly, the contrast of interfere fringes also gradually decreased and completely disappeared after 3 h, which indicates that the reflectivity of the splicing face between the SMF and PCF gradually reduced. This was very likely induced by the gradual softening of the doped core of the SMF rather than the pure fused silica PCF cavity. Nevertheless, since the measuring error increases at a slower rate of ~8.7 °C/h in the first 30 min and the contrast is still clear enough, the sensor was capable of operating at temperatures up to 1200 °C in the short term (<30 min) with acceptable precision. It can also be obtained from the stability test that the measurement accuracy is strongly related to measurement time and temperature. Assuming that it takes 10 min to complete the measurement, the minimum temperature uncertainty at 1100 °C and 1200 °C is ~0.12 °C and ~1.45 °C, respectively.

## 5. Conclusions

In summary, we have fabricated and demonstrated an F-P interferometric high-temperature sensor based on a silica glass solid-core photonic crystal fiber with a central air-bore. By splicing a stub of photonic crystal fiber to a standard single-mode fiber, an intrinsic Fabry-Perot cavity was formed inside the photonic crystal fiber. In a wide measurement range from 300 to 1200 °C, the sensor has a high sensitivity of 15.61 pm/°C with a linearity of 99.76%. The stability test shows that the sensor can work stably under 1100 °C for a consecutive 24 h, and the short-term operation temperature can reach 1200 °C. The presented interferometric sensor is compact in size and has good performance indices, such as an extended working range and high sensitivity. To our knowledge, this sensor has an upper limit and sensitivity superior to many reported interferometric optical fiber temperature sensors. In the future, we will attempt to connect the PCF sensor head to pure silica fibers (without using of doped SMF), which is expected to further increase the maximum operation temperature.
